# Analysis of Anxiety and Depression Status and Risk Factors in Postmenopausal Women With Diabetes Mellitus Complicated by Hypothyroidism

**DOI:** 10.62641/aep.v54i1.2119

**Published:** 2026-02-15

**Authors:** Yan Hong, Daoyun Xu, Jingya Chen, Ying Hu, Ning Wang, Xiaoran Liu

**Affiliations:** ^1^School of Medicine, Huainan Union University, 232038 Huainan, Anhui, China; ^2^Department of Emergency, Huainan Chaoyang Hospital, 232038 Huainan, Anhui, China

**Keywords:** postmenopausal women, type 2 diabetes mellitus, hypothyroidism, depression, anxiety

## Abstract

**Background:**

This study aimed to investigate the prevalence of anxiety and depression symptoms and analyse the associated risk factors in postmenopausal women with type 2 diabetes mellitus (T2DM) and comorbid hypothyroidism.

**Methods:**

A cross-sectional study design was employed, enrolling 152 postmenopausal women with T2DM and hypothyroidism who attended Huainan Chaoyang Hospital between February 2024 and August 2025. Psychological status was assessed using the Hamilton Depression Rating Scale and the Hamilton Anxiety Rating Scale. Demographic characteristics, clinical features and laboratory parameters were collected.

**Results:**

Amongst the 152 patients, the prevalence rates of depressive and anxiety symptoms were 26.97% and 34.87%, respectively. Between-group analyses showed that the depression group had significantly longer durations of T2DM and hypothyroidism and higher glycosylated haemoglobin (HbA1c) and thyroid-stimulating hormone (TSH) levels than the non-depression group. The anxiety group was significantly younger than the non-anxiety group, with longer T2DM duration and higher TSH levels (*p* < 0.05). Multivariate logistic regression analysis identified increased HbA1c level (Odds Ratio [OR] = 1.43), increased TSH level (OR = 1.36) and longer T2DM duration (OR = 1.21) as independent risk factors for depressive symptoms, whereas higher income served as a protective factor (OR = 0.19). For anxiety symptoms, younger age (OR = 0.88), longer T2DM duration (OR = 1.19) and increased TSH (OR = 1.23) were independent risk factors.

**Conclusions:**

Anxiety and depressive symptoms are prevalent amongst postmenopausal women with T2DM and hypothyroidism. Poor glycaemic control, thyroid dysfunction and longer diabetes duration are primary risk factors.

## Introduction

Type 2 diabetes mellitus (T2DM) represents a highly prevalent chronic non-communicable disease in China and globally, posing a serious threat to public health [1, 2]. According to recent reports, approximately 588 million adults worldwide were affected by diabetes in 2024, with projections indicating an increase to 852 million by 2050 [1]. As a metabolic disorder, the effect of T2DM extends beyond abnormal glucose metabolism and various complications to encompass close associations with mental health issues. Studies have demonstrated that patients with T2DM face a significantly increased risk of developing depression and anxiety compared with the general population [3]. These psychological disorders can reduce treatment adherence, lead to poor glycaemic control, increase complication risks and ultimately affect long-term survival.

Hypothyroidism constitutes another common endocrine disorder characterised primarily by insufficient synthesis or secretion of thyroid hormones, showing notably higher prevalence amongst female patients, particularly older women [4, 5]. Existing evidence indicates a certain correlation between diabetes and hypothyroidism, with a higher incidence of thyroid dysfunction observed in diabetic populations [6]. Research indicates that 26.5% of patients with T2DM exhibit thyroid dysfunction, with 84.9% of these cases being hypothyroidism [7]. Furthermore, hypothyroidism not only exacerbates metabolic disturbances and insulin resistance but also demonstrates strong associations with depression and anxiety [8]. A study from Iran reported that over 50% of patients with hypothyroidism experience anxiety and depression [9].

Postmenopausal women represent a distinct population group characterised by rapid declines in oestrogen levels, accompanied by lipid metabolism disorders, bone loss and altered immune responses [10]. Research indicates that decreased oestrogen levels may promote the development of autoimmune diseases, thereby increasing the risk of thyroid disorders [11]. Simultaneously, menopause itself shows strong correlations with psychological disturbances, as evidenced by significantly increased rates of depression and anxiety amongst postmenopausal women compared with premenopausal counterparts [12]. Consequently, postmenopausal women are at a vulnerable stage for diabetes and hypothyroidism whilst being more susceptible to psychological disorders due to endocrine changes, collectively constituting a high-risk population.

The effect of these two chronic conditions on mental health may not be a simple summation, but rather exhibit synergistic effects. Abdul Jaffar Azad and Zohara [13] indicated that thyroid hormones regulate the synthesis and release of neurotransmitters, thereby influencing mood and cognitive function. Meanwhile, diabetes induces metabolic disorders and oxidative stress, which similarly disrupt neurotransmitter balance. When these two conditions coexist, they indirectly exacerbate adverse effects on mental health by jointly impairing neurological function. Additionally, hypothyroidism may further deteriorate insulin resistance and dyslipidaemia, and diabetes accelerates vascular and neural damage. Their combined action could lead to structural and functional impairments in the central nervous system, thereby increasing the risk of mood disorders and cognitive impairment [14, 15]. Therefore, the comorbidity of diabetes and hypothyroidism in postmenopausal women presents not only metabolic and endocrine challenges but also a crucial focus in mental health management.

However, despite existing literature confirming correlations between either diabetes or hypothyroidism with depression and anxiety, research on mental health in postmenopausal women with comorbid diabetes and hypothyroidism remains limited. Comprehensive investigations into risk factors, including thyroid-related parameters, diabetes duration, glycaemic control levels and complication burden, are particularly scarce. Therefore, an exploratory study was designed to investigate the current status of anxiety and depression and explore the relevant risk factors in postmenopausal women with diabetes and hypothyroidism, hoping to provide scientific evidence for developing early screening and comprehensive intervention strategies in clinical practice.

## Methods

### Study Population

A cross-sectional study design was employed. The study cohort consisted of 152 postmenopausal women with concurrent T2DM and hypothyroidism, who were recruited from Huainan Chaoyang Hospital between February 2024 and August 2025. The study protocol was approval by the Ethics Committee of Huainan Chaoyang Hospital (PJ2024-L013). Written informed consent was obtained from all participants prior to enrolment, and the study was conducted in accordance with the principles outlined in the Declaration of Helsinki.

The inclusion criteria were as follows: female sex; aged ≥45 years; meeting the criteria for postmenopausal status (12 consecutive months of amenorrhoea, corroborated by follicle-stimulating hormone levels in routine admission tests, excluding pathological or surgical causes) [10]; prior established diagnosis of T2DM [16]; previous diagnosis of hypothyroidism, encompassing overt and subclinical forms, and receipt of appropriate medical management for this condition [17]; and capacity to complete the questionnaires and provide voluntary informed consent. Overt hypothyroidism is defined by increased serum thyrotropin concentrations (>10.0 mIU/L) accompanied with reduced serum free thyroxine concentrations (<12.0 pmol/L). Subclinical hypothyroidism is defined as increased serum TSH concentration (>4.2 mIU/L), with serum FT4 levels within the normal reference range (12.0–22.0 pmol/L).

Participants were excluded on the basis of the following criteria: history of thyroid tumours, prior thyroidectomy or radioactive iodine therapy; pre-existing diagnosis of depressive disorders, anxiety disorders or other psychiatric conditions; taking antidepressants or anti-anxiety medication; presence of severe acute or chronic somatic diseases (e.g., acute myocardial infarction, severe hepatic or renal dysfunction and malignancy); current pregnancy or lactation, or amenorrhea due to other identifiable causes; and inability to comprehend or cooperate with the questionnaire assessment.

A post-hoc power analysis was conducted to assess the adequacy of sample size for multivariate analysis. With the primary outcome of depressive symptoms (prevalence of 27%) as an example, α = 0.05, statistical power (1-β) = 80% and R^2^ = 0.15, the analysis indicated that the current sample size (n = 152) was sufficient to detect an Odds Ratio (OR) ≥2.0 in multivariate logistic regression. Concurrently, the number of events for the primary outcomes (depression n = 41, anxiety n = 53) satisfied the empirical guideline requiring at least 10 events per parameter to be estimated in logistic regression analysis.

### Assessment Scales

This study assessed patients’ anxiety and depression levels through interviews conducted by professionally trained personnel following admission to hospital. The assessment instruments comprised the 17-item Hamilton Depression Rating Scale (HAMD) and the 14-item Hamilton Anxiety Rating Scale (HAMA) [18, 19]. A score of ≥10 on HAMD was defined as indicative of depressive symptoms, and a cut-off score of ≥14 on HAMA was established for identifying anxiety symptoms [20, 21]. The Chinese versions of both scales demonstrated good internal consistency in populations with depression and anxiety disorders [22, 23]. Completion of HAMD-17 or HAMA-14 assessment requires 5–10 min. All assessments were conducted in a quiet, private environment to ensure data quality.

### Data Collection

Demographic and sociological information, including age, place of residence, living alone status, educational level, household income and history of smoking and alcohol consumption, was obtained by trained research personnel through standardised questionnaires supplemented by medical record review. Disease-related information, encompassing duration of T2DM, duration and aetiology of hypothyroidism and presence of diabetic complications, was extracted from electronic medical records. Laboratory parameters consisting of fasting blood glucose (FBG), glycated haemoglobin (HbA1c), thyroid-stimulating hormone (TSH), free triiodothyronine (FT3) and free thyroxine (FT4) were derived from the initial laboratory tests following hospital admission, as exported from the hospital’s laboratory information system. Psychological assessments using the HAMD and HAMA scales were conducted in person by researchers at the time of patient admission. For the purpose of this study, diabetic complications only included clinically confirmed, relatively severe complications. Diabetic kidney disease was defined as estimated glomerular filtration rate <60 mL/min/1.73 m^2^ or urinary albumin-to-creatinine ratio ≥300 mg/g [24]. Diabetic retinopathy required confirmation through fundoscopic examination, including non-proliferative and proliferative stages. Diabetic neuropathy was defined by the presence of characteristic clinical symptoms or signs, corroborated by peripheral nerve electrophysiological studies. Patients may present with multiple concurrent complications, and the presence of complications was determined by the fulfilment of the criterion that ‘at least one clinically confirmed severe complication is present’.

All psychological assessments, clinical measurements and laboratory sampling for the subjects in this study were completed concurrently within 24 h of hospital admission. The procedure strictly adhered to the sequence of ‘first collecting baseline information and laboratory samples, followed by psychological assessment’, with no temporal discrepancies.

### Statistical Analysis

All data were analysed using SPSS Statistics (version 26.0, IBM, Armonk, NY, USA). The normality of continuous variables was assessed using Kolmogorov–Smirnov test. Normally and non-normally distributed variables were expressed as mean ± standard deviation and median (interquartile range), respectively. Comparisons between groups were performed using independent sample *t*-test or Mann–Whitney U test. Categorical variables were summarised as counts and percentages, with group comparisons performed using chi-square tests or Fisher’s exact test, as appropriate. Univariate and multivariate logistic regression analyses were employed to identify risk factors, with anxiety and depression serving as dependent variables, respectively. Clinically relevant and demographic characteristics were included as independent variables in these models. Pearson’s correlation analysis was applied to examine associations between selected continuous variables. A two-tailed *p* < 0.05 was considered statistically significant for all analyses.

## Results

### Baseline Characteristics and Laboratory Findings

A total of 152 postmenopausal women with T2DM and hypothyroidism were included in this study. The detailed baseline demographic and clinical characteristics of all participants are presented in Table 1. The mean age of the patients was 51.41 years, with a mean body mass index (BMI) of 21.39 kg/m^2^. Regarding educational attainment, the majority (72.37%) had an educational level of junior high school or below. In terms of household income, the highest proportion of patients (42.11%) reported a monthly household income per capita ranging from 3001 to 5000 Chinese yuan. Autoimmune thyroiditis was the predominant aetiology of hypothyroidism (80.92%). More than half of the participants resided in rural areas (57.24%), and most did not live alone (63.16%). Furthermore, 37.50% and 26.97% of patients reported a history of smoking and alcohol consumption, respectively. Diabetic complications were present in 55.26% of the patients.

**Table 1.** **Baseline demographics of patients**.

Variables		Total (n = 152)
Age (years), mean ± SD		51.41 ± 3.31
BMI (kg/m^2^), mean ± SD		21.39 ± 2.36
Duration of hypothyroidism, M (Q_1_, Q_3_)		4 (3, 5)
Duration of T2DM, M (Q_1_, Q_3_)		7 (5, 8)
Educational level, n (%)		
	Primary school	39 (25.66)
	Junior high school	71 (46.71)
	High school	32 (21.05)
	College or University	10 (6.58)
Monthly household income per capita (CNY), n (%)		
	≤3000	53 (34.87)
	3001–5000	64 (42.11)
	>5000	35 (23.03)
Aetiology of hypothyroidism, n (%)		
	Autoimmune thyroiditis	123 (80.92)
	Other	29 (19.08)
Residence, n (%)		
	Rural	87 (57.24)
	Urban	65 (42.76)
Living alone, n (%)		
	No	96 (63.16)
	Yes	56 (36.84)
Smoking, n (%)		
	No	95 (62.50)
	Yes	57 (37.50)
Alcohol consumption, n (%)		
	No	111 (73.03)
	Yes	41 (26.97)
T2DM complications, n (%)		
	No	68 (44.74)
	Yes	84 (55.26)

BMI, body mass index; T2DM, type 2 diabetes mellitus. Other: central hypothyroidism, post-thyroidectomy/post-iodine-131 therapy hypothyroidism, drug-induced hypothyroidism and cases of unknown aetiology. Exchange rate: 1 USD = 6.97 CNY.

### Laboratory Investigations and Scale Assessment Results

The laboratory test results and scale assessment scores for all patients are summarised in Table 2. The metabolic indicators revealed a median FBG of 8.6 mmol/L and a median HbA1c of 8.5%. Regarding thyroid function, the mean TSH level was 7.40 mIU/L, with FT3 at 5.31 pmol/L and FT4 at 15.19 pmol/L, indicating overall poor glycaemic control amongst patients. The psychological assessment results revealed a median HAMA score of 13 and a median HAMD score of 8, suggesting that this cohort bears a certain degree of anxiety and depressive symptom burden.

**Table 2.** **Laboratory findings and scale scores of patients**.

Variables	Total (n = 152)
FBG (mmol/L), M (Q_1_, Q_3_)	8.6 (6.6, 12.2)
HbA1c (%), M (Q_1_, Q_3_)	8.5 (7, 10)
TSH (mIU/L), mean ± SD	7.40 ± 2.23
FT3 (pmol/L), mean ± SD	5.31 ± 1.19
FT4 (pmol/L), mean ± SD	15.19 ± 4.64
HAMA, M (Q_1_, Q_3_)	13 (9, 15)
HAMD, M (Q_1_, Q_3_)	8 (5, 10)

FBG, fasting blood glucose; HbA1c, glycated haemoglobin; TSH, thyroid-stimulating hormone; FT3, free triiodothyronine; FT4, free thyroxine; HAMA, Hamilton Anxiety Rating Scale; HAMD, Hamilton Depression Rating Scale; SD, Standard Deviation.

### Comparison of Clinical Characteristics Between Depression and Non-Depression Groups

On the basis of the HAMD scores, 41 of the 152 patients were classified into the depression group, whereas 111 comprised the non-depression group. A detailed comparison of characteristics between the two groups is presented in Table 3. The results indicated no statistically significant differences between groups regarding age, BMI, residence, living alone status, aetiology of hypothyroidism, educational level, smoking status, alcohol consumption, diabetic complications, FT3, FT4 and HAMA scores (*p* > 0.05). However, significant differences emerged in disease duration, metabolic parameters and key thyroid function indicators. The depression group demonstrated significantly longer durations of hypothyroidism and T2DM than the non-depression group. Regarding metabolic control, the depression group exhibited significantly higher HbA1c levels, with FBG showing a trend toward higher values. Most notably, the TSH levels in the depression group substantially increased compared with that in the non-depression group (*p* < 0.001). Although the intergroup comparison of income levels did not reach statistical significance, the proportion of low-income patients was higher in the depression group.

**Table 3.** **Comparative analysis of differences between patients with and without depression**.

Variables		Non-depression (n = 111)	Depression (n = 41)	Statistic	*p*	Cohen’s d
Age (years), mean ± SD		51.60 ± 3.39	50.88 ± 3.06	t = 1.20	0.231	–0.22
BMI (kg/m^2^), mean ± SD		21.21 ± 2.24	21.88 ± 2.62	t = –1.56	0.122	0.28
Duration of hypothyroidism, mean ± SD		3.63 ± 1.37	4.46 ± 1.72	t = –3.10	0.002	0.54
Duration of T2DM, mean ± SD		6.37 ± 2.61	7.73 ± 2.65	t = –2.84	0.005	0.52
FBG (mmol/L), mean ± SD		9.10 ± 3.40	10.22 ± 3.24	t = –1.83	0.069	0.34
HbA1c (%), mean ± SD		8.16 ± 1.76	9.29 ± 2.18	t = –2.98	0.004	0.59
TSH (mIU/L), mean ± SD		6.98 ± 2.13	8.54 ± 2.12	t = –3.99	<0.001	0.74
FT3 (pmol/L), mean ± SD		5.26 ± 1.18	5.46 ± 1.22	t = –0.93	0.355	0.17
FT4 (pmol/L), mean ± SD		15.60 ± 4.84	14.06 ± 3.89	t = 1.83	0.069	–0.35
HAMA, mean ± SD		12.57 ± 4.93	13.56 ± 6.63	t = –0.87	0.385	0.17
HAMD, mean ± SD		6.44 ± 2.31	13.24 ± 3.06	t = –12.93	<0.001	2.52
Educational level, n (%)				χ^2^ = 1.22	0.748	
	Primary school	31 (27.93)	8 (19.51)			
	Junior high school	51 (45.95)	20 (48.78)			
	High school	22 (19.82)	10 (24.39)			
	College or university	7 (6.31)	3 (7.32)			
Monthly household income per capita (CNY), n (%)				χ^2^ = 5.24	0.073	
	≤3000	33 (29.73)	20 (48.78)			
	3001–5000	49 (44.14)	15 (36.59)			
	>5000	29 (26.13)	6 (14.63)			
Aetiology of hypothyroidism, n (%)				χ^2^ = 0.30	0.584	
	Autoimmune thyroiditis	91 (81.98)	32 (78.05)			
	Other	20 (18.02)	9 (21.95)			
Residence, n (%)				χ^2^ = 0.04	0.844	
	Rural	63 (56.76)	24 (58.54)			
	Urban	48 (43.24)	17 (41.46)			
Living alone, n (%)				χ^2^ = 2.18	0.140	
	No	74 (66.67)	22 (53.66)			
	Yes	37 (33.33)	19 (46.34)			
Smoking, n (%)				χ^2^ = 0.06	0.813	
	No	70 (63.06)	25 (60.98)			
	Yes	41 (36.94)	16 (39.02)			
Alcohol consumption, n (%)				χ^2^ = 0.00	0.981	
	No	81 (72.97)	30 (73.17)			
	Yes	30 (27.03)	11 (26.83)			
T2DM complications, n (%)				χ^2^ = 2.55	0.111	
	No	54 (48.65)	14 (34.15)			
	Yes	57 (51.35)	27 (65.85)			

T2DM, type 2 diabetes mellitus; BMI, body mass index; FBG, fasting blood glucose; HbA1c, glycated haemoglobin; TSH, thyroid-stimulating hormone; FT3, free triiodothyronine; FT4, free thyroxine; HAMA, Hamilton Anxiety Rating Scale; HAMD, Hamilton Depression Rating Scale; SD, Standard Deviation. Exchange rate: 1 USD = 6.97 CNY.

### Logistic Regression Analysis for Depressive Symptoms

Univariate and multivariate logistic regression analyses were performed to identify factors associated with depressive symptoms, with results detailed in Tables 4,5. The univariate analysis identified longer duration of hypothyroidism (OR = 1.46), longer T2DM duration (OR = 1.22), increased HbA1c (OR = 1.38) and higher TSH levels (OR = 1.41) as risk factors for depressive symptoms. Additionally, higher monthly household income per capita demonstrated a protective effect, reducing the risk of depression to 0.34 times that of the low-income group (OR = 0.34). The FBG and FT4 levels showed statistical trends toward association with depression risk, without reaching significance.

**Table 4.** **Univariate logistic regression for depression**.

Variables		β	*p*	OR (95% CI)
Age (years)		–0.07	0.230	0.94 (0.84–1.04)
BMI (kg/m^2^)		0.12	0.123	1.13 (0.97–1.32)
Duration of hypothyroidism		0.38	0.003	1.46 (1.13–1.88)
Duration of T2DM		0.20	0.007	1.22 (1.06–1.41)
Educational level				
	Primary school			1.00 (Reference)
	Junior high school	0.42	0.380	1.52 (0.60–3.87)
	High school	0.57	0.304	1.76 (0.60–5.18)
	College or university	0.51	0.524	1.66 (0.35–7.90)
Monthly household income per capita (CNY), n (%)				
	≤3000			1.00 (Reference)
	3001–5000	–0.68	0.095	0.51 (0.23–1.13)
	>5000	–1.07	0.043	0.34 (0.12–0.97)
Aetiology of hypothyroidism				
	Autoimmune thyroiditis			1.00 (Reference)
	Other	0.25	0.584	1.28 (0.53–3.10)
Residence				
	Rural			1.00 (Reference)
	Urban	–0.07	0.844	0.93 (0.45–1.92)
Living alone				
	No			1.00 (Reference)
	Yes	0.55	0.142	1.73 (0.83–3.58)
Smoking				
	No			1.00 (Reference)
	Yes	0.09	0.814	1.09 (0.52–2.28)
Alcohol consumption				
	No			1.00 (Reference)
	Yes	–0.01	0.981	0.99 (0.44–2.22)
T2DM complications				
	No			1.00 (Reference)
	Yes	0.60	0.113	1.83 (0.87–3.85)
FBG		0.10	0.072	1.10 (0.99–1.23)
HbA1c		0.32	0.002	1.38 (1.13–1.69)
TSH		0.35	<0.001	1.41 (1.17–1.71)
FT3		0.14	0.353	1.15 (0.85–1.56)
FT4		–0.07	0.072	0.93 (0.86–1.01)

BMI, body mass index; T2DM, type 2 diabetes mellitus; FBG, fasting blood glucose; HbA1c, glycated haemoglobin; TSH, thyroid-stimulating hormone; FT3, free triiodothyronine; FT4, free thyroxine; OR, Odds Ratio; CI, Confidence Interval. Exchange rate: 1 USD = 6.97 CNY.

**Table 5.** **Multivariate logistic regression for depression**.

Variables		β	*p*	OR (95% CI)
Duration of hypothyroidism		0.52	0.081	1.19 (0.98–1.37)
Duration of T2DM		0.19	0.029	1.21 (1.02–1.44)
Monthly household income per capita (CNY), n (%)				
	≤3000			1.00 (Reference)
	3001–5000	–0.95	0.065	0.39 (0.14–1.06)
	>5000	–1.64	0.011	0.19 (0.05–0.68)
HbA1c		0.36	0.004	1.43 (1.12–1.82)
TSH		0.31	0.005	1.36 (1.10–1.69)

HbA1c, glycated haemoglobin; TSH, thyroid-stimulating hormone; T2DM, type 2 diabetes mellitus; OR, Odds Ratio; CI, Confidence Interval. Exchange rate: 1 USD = 6.97 CNY.

The multivariate logistic regression analysis, after adjusting for variables showing statistical significance or trends in the univariate analysis, confirmed increased HbA1c (OR = 1.43) and higher TSH levels (OR = 1.36) as independent risk factors for depressive symptoms. Longer T2DM duration remained significantly associated with increased depression risk (OR = 1.21). Furthermore, monthly household income per capita >5000 CNY (1 USD = 6.97 CNY) was identified as an independent protective factor against depressive symptoms (OR = 0.19).

### Comparison of Clinical Characteristics Between Anxiety and Non-Anxiety Groups

In accordance with the HAMA scores, 53 of the 152 patients were categorised into the anxiety group, whereas 99 constituted the non-anxiety group. The clinical characteristics of both groups are compared in Table 6. The results revealed that the anxiety group was significantly younger than the non-anxiety group. Regarding disease characteristics, the anxiety group demonstrated significantly longer duration of T2DM and higher TSH levels. The FT3 levels were marginally lower in the anxiety group, with this difference approaching statistical significance. However, no statistically significant differences were observed between the two groups in terms of BMI, FBG, HbA1c, FT4, HAMD scores or any of the collected sociodemographic factors (including educational level, income, residence and living alone status) and lifestyle factors (smoking and alcohol consumption, *p* > 0.05). The prevalence of diabetic complications, the duration of hypothyroidism and the aetiology of hypothyroidism showed no significant difference between the groups.

**Table 6.** **Comparative analysis of differences between patients with and without anxiety**.

Variables		Non-anxiety group (n = 99)	Anxiety group (n = 53)	Statistic	*p*	Cohen’s d
Age (years), mean ± SD		51.88 ± 3.34	50.53 ± 3.08	t = 2.44	0.016	–0.42
BMI (kg/m^2^), mean ± SD		21.39 ± 2.36	21.40 ± 2.38	t = –0.01	0.991	0.00
Duration of hypothyroidism, mean ± SD		3.73 ± 1.48	4.09 ± 1.55	t = –1.43	0.154	0.24
Duration of T2DM, mean ± SD		6.31 ± 2.70	7.53 ± 2.49	t = –2.72	0.007	0.47
FBG (mmol/L), mean ± SD		9.50 ± 3.30	9.22 ± 3.55	t = 0.50	0.617	–0.08
HbA1c (%), mean ± SD		8.48 ± 1.91	8.43 ± 2.00	t = 0.15	0.878	–0.03
TSH (mIU/L), mean ± SD		7.03 ± 2.23	8.10 ± 2.07	t = –2.89	0.004	0.50
FT3 (pmol/L), mean ± SD		5.45 ± 1.14	5.06 ± 1.24	t = 1.95	0.053	–0.33
FT4 (pmol/L), mean ± SD		15.51 ± 4.71	14.59 ± 4.49	t = 1.16	0.246	–0.20
HAMA, mean ± SD		9.89 ± 3.41	18.34 ± 4.05	t = –13.62	<0.001	2.27
HAMD, mean ± SD		8.39 ± 3.88	8.06 ± 4.09	t = 0.50	0.617	–0.08
Educational level, n (%)				χ^2^ = 1.44	0.695	
	Primary school	23 (23.23)	16 (30.19)			
	Junior high school	47 (47.47)	24 (45.28)			
	High school	23 (23.23)	9 (16.98)			
	College or university	6 (6.06)	4 (7.55)			
Monthly household income per capita (CNY), n (%)				χ^2^ = 1.35	0.508	
	≤3000	33 (33.33)	20 (37.74)			
	3001–5000	45 (45.45)	19 (35.85)			
	>5000	21 (21.21)	14 (26.42)			
Aetiology of hypothyroidism, n (%)				χ^2^ = 0.00	0.961	
	Autoimmune thyroiditis	80 (80.81)	43 (81.13)			
	Other	19 (19.19)	10 (18.87)			
Residence, n (%)				χ^2^ = 2.22	0.136	
	Rural	61 (61.62)	26 (49.06)			
	Urban	38 (38.38)	27 (50.94)			
Living alone, n (%)				χ^2^ = 2.55	0.110	
	No	58 (58.59)	38 (71.70)			
	Yes	41 (41.41)	15 (28.30)			
Smoking, n (%)				χ^2^ = 0.09	0.758	
	No	61 (61.62)	34 (64.15)			
	Yes	38 (38.38)	19 (35.85)			
Alcohol consumption, n (%)				χ^2^ = 0.78	0.379	
	No	70 (70.71)	41 (77.36)			
	Yes	29 (29.29)	12 (22.64)			
T2DM complications, n (%)				χ^2^ = 0.86	0.353	
	No	47 (47.47)	21 (39.62)			
	Yes	52 (52.53)	32 (60.38)			

BMI, body mass index; FBG, fasting blood glucose; HbA1c, glycated haemoglobin; TSH, thyroid-stimulating hormone; FT3, free triiodothyronine; FT4, free thyroxine; HAMA, Hamilton Anxiety Rating Scale; HAMD, Hamilton Depression Rating Scale; T2DM, type 2 diabetes mellitus; SD, Standard Deviation. Exchange rate: 1 USD = 6.97 CNY.

### Logistic Regression Analysis for Anxiety Symptoms

The univariate logistic regression analysis of factors influencing anxiety symptoms (Table 7) identified younger age (OR = 0.88), longer T2DM duration (OR = 1.19) and higher TSH levels (OR = 1.26) as significant risk factors for anxiety symptoms. Furthermore, lower FT3 levels showed a marginal association with increased anxiety risk (OR = 0.75). These variables with *p* < 0.05 in the univariate analysis were subsequently included in a multivariate logistic regression model for adjustment. As shown in Table 8, younger age (OR = 0.88), longer T2DM duration (OR = 1.19) and higher TSH levels (OR = 1.23) were confirmed as independent risk factors for anxiety symptoms.

**Table 7.** **Univariate logistic regression for anxiety**.

Variables		β	*p*	OR (95% CI)
Age (years)		–0.13	0.018	0.88 (0.79–0.98)
BMI (kg/m^2^)		0.00	0.991	1.00 (0.87–1.15)
Duration of hypothyroidism		0.16	0.155	1.18 (0.94–1.47)
Duration of T2DM		0.18	0.009	1.19 (1.04–1.37)
Educational level				
	Primary school			1.00 (Reference)
	Junior high school	–0.31	0.452	0.73 (0.33–1.64)
	High school	–0.58	0.260	0.56 (0.21–1.53)
	College or university	–0.04	0.953	0.96 (0.23–3.95)
Monthly household income per capita (CNY)				
	≤3000			1.00 (Reference)
	3001–5000	–0.36	0.359	0.70 (0.32–1.51)
	>5000	0.10	0.831	1.10 (0.46–2.64)
Aetiology of hypothyroidism				
	Autoimmune thyroiditis			1.00 (Reference)
	Other	–0.02	0.961	0.98 (0.42–2.29)
Residence				
	Rural			1.00 (Reference)
	Urban	0.51	0.137	1.67 (0.85–3.27)
Living alone				
	No			1.00 (Reference)
	Yes	–0.58	0.112	0.56 (0.27–1.15)
Smoking				
	No			1.00 (Reference)
	Yes	–0.11	0.758	0.90 (0.45–1.79)
Alcohol consumption				
	No			1.00 (Reference)
	Yes	–0.35	0.380	0.71 (0.33–1.53)
T2DM complications				
	No			1.00 (Reference)
	Yes	0.32	0.354	1.38 (0.70–2.71)
FBG		–0.03	0.615	0.97 (0.88–1.08)
HbA1c		–0.01	0.877	0.99 (0.83–1.17)
TSH		0.23	0.006	1.26 (1.07–1.48)
FT3		–0.29	0.055	0.75 (0.56–1.01)
FT4		–0.04	0.245	0.96 (0.89–1.03)

BMI, body mass index; FBG, fasting blood glucose; HbA1c, glycated haemoglobin; TSH, thyroid-stimulating hormone; FT3, free triiodothyronine; FT4, free thyroxine; HAMA, Hamilton Anxiety Rating Scale; HAMD, Hamilton Depression Rating Scale; T2DM, type 2 diabetes mellitus; OR, Odds Ratio; CI, Confidence Interval. Exchange rate: 1 USD = 6.97 CNY.

**Table 8.** **Multivariate logistic regression for anxiety**.

Variables	β	*p*	OR (95% CI)
Age (years)	–0.13	0.028	0.88 (0.79–0.99)
Duration of T2DM	0.17	0.017	1.19 (1.03–1.37)
TSH	0.21	0.017	1.23 (1.04–1.47)

T2DM, type 2 diabetes mellitus; TSH, thyroid-stimulating hormone; OR, Odds Ratio.

## Discussion

This study focused on postmenopausal women with T2DM and comorbid hypothyroidism, investigating the prevalence of anxiety and depressive symptoms and exploring the associated metabolic, endocrine and sociodemographic risk factors. The prevalence rates of depressive and anxiety symptoms in this patient cohort were 26.97% and 34.87% respectively. Specifically, increased TSH levels, longer T2DM duration and poorer glycaemic control were identified as independent risk factors for depressive symptoms, whereas higher household income demonstrated a protective effect. For anxiety symptoms, besides associations with higher TSH levels and longer T2DM duration, younger age emerged as an additional risk factor [25]. These findings collectively suggest that the development of mood disorders in postmenopausal women with T2DM and hypothyroidism is closely associated with endocrine dysregulation, suboptimal metabolic control and specific sociodemographic characteristics [26, 27].

This study identified TSH level increase as a common and independent risk factor for depressive and anxiety symptoms. Thyroid hormone deficiency may impair emotional regulation by disrupting the synthesis and metabolism of central nervous system neurotransmitters, a mechanism consistent with existing literature reports [28, 29]. Similarly, Liu *et al*. [30] reported higher TSH levels in populations with increased depression scores, and Maier *et al*. [21] demonstrated positive correlations between TSH levels and HAMA and HAMD scores in patients with hypothyroidism. Although Wu *et al*. [31] found no significant difference in the TSH levels between healthy controls and patients with autoimmune diseases, they observed a significant positive correlation between TSH levels and HAMD scores. The multivariate regression in the present study supports this association, suggesting that TSH may serve not only as a diagnostic marker for hypothyroidism but also as an effective warning indicator for mental health risks in this population. A notable detail that the relationship between TSH levels and emotional state may be complex. However, other studies [9] failed to identify a significant association between TSH levels and anxiety/depression. This discrepancy may stem from differences in study populations (such as the aetiology of hypothyroidism or disease activity), variations in psychological assessment tools or limitations in sample size. Future research with more homogeneous cohorts is required to clarify the precise role of TSH in emotional disorders.

An equally noteworthy finding of this investigation is the identification of decreased FT3 levels as an independent risk factor for anxiety symptoms. As the biologically active form of thyroid hormone in peripheral tissues, FT3 may exert more direct effects on the central nervous system than TSH [4, 32]. Research indicated that thyroid hormones play crucial roles in hippocampal neuronal survival, synaptic plasticity and cognitive function, with deficiency directly contributing to emotional regulation impairments [33]. The findings of the present study extend this mechanistic understanding to anxiety symptomatology, suggesting that FT3 levels may provide critical information beyond TSH measurements when evaluating patients’ mental health. This observation offers at least partial explanation for the presence of significant emotional disturbances in some patients with subclinical hypothyroidism, where relative FT3 deficiency may play a pivotal role.

This study confirmed that longer T2DM duration and increased HbA1c levels serve as independent predictors of depressive symptoms. As a chronic condition, the extended disease course of diabetes creates an ongoing psychological burden through treatment demands and apprehension about potential complications [34]. Concurrently, the suboptimal long-term glycaemic control reflected by increased HbA1c is associated with increased risk of mood disorders of developing mood disorders [35]. Persistent hyperglycaemic state can trigger systemic microinflammatory conditions, enhanced oxidative stress and vascular endothelial dysfunction-pathological processes that collectively compromise blood–brain barrier integrity, impair cerebral blood flow and directly damage neurons, thereby contributing to the pathogenesis of anxiety disorders [36]. The findings of the present study established a robust association between HbA1c and depression in menopausal women with comorbid T2DM and hypothyroidism, underscoring the critical importance of optimising long-term glycaemic control. However, a notable detail that this study did not identify a significant relationship between FBG levels and either anxiety or depression, whereas previous research has suggested potential associations between this parameter and mood disorders [37]. This discrepancy may be attributed to the substantial glycaemic variability in patients with T2DM, which may attenuate potential differences, thereby highlighting the necessity for subsequent investigations with larger sample sizes.

Regarding sociodemographic factors, the findings align with expectations in demonstrating that higher per capita household income serves as a protective factor against depressive symptoms. This association likely stems from enhanced economic conditions facilitating improved healthcare access, reduced financial burden of treatment, healthier lifestyle choices and enhanced social security, all of which may buffer the psychological stress associated with chronic diseases [38, 39]. This observation highlights the clinical importance of providing enhanced psychosocial support to patients from lower socioeconomic backgrounds. Furthermore, a particularly intriguing finding emerged regarding the association between younger age and increased anxiety risk within the study population. This appears counterintuitive given the conventional understanding that younger age typically correlates with greater psychological resilience [40]. One plausible explanation is that the early onset of two chronic conditions (T2DM and hypothyroidism) during menopausal transition may generate heightened concerns about future health status. Additionally, relatively younger middle-aged patients are likely navigating peak career and family responsibilities, creating a conflict between disease management demands and developmental tasks that potentially amplifies anxiety experiences. This finding is not isolated because previous investigations have reported similar phenomena [9, 31]. Furthermore, the potential for certain biases must be considered. For instance, survival bias may result in elderly patients with severe comorbidities failing to survive until study inclusion. Simultaneously, differences in healthcare-seeking behaviour across age groups may exist, with younger patients potentially being more sensitive to psychological distress and more inclined to report symptoms, thereby being more frequently identified within the attending population.

The mean BMI of patients in this study was lower than that reported for the general T2DM population. This phenomenon may be attributable to the following combined factors: Firstly, the study population comprised postmenopausal women all with hypothyroidism, where the condition and its treatment may have influenced body weight. Secondly, as hospitalised patients, this cohort may represent a subgroup with poorer glycaemic control, longer disease duration, or a catabolic state. Finally, the study may have included a higher proportion of ‘lean diabetics’, whose pathophysiology may be more centred on insufficient insulin secretion rather than insulin resistance. This characteristic implies that the findings regarding the association between metabolic factors and psychological symptoms in this study may be more applicable to non-obese female T2DM patients with comorbid hypothyroidism. Caution is warranted when extrapolating conclusions to the typical T2D population characterised primarily by obesity. Future research could compare the relationship amongst diabetes, hypothyroidism and mental health across different BMI subtypes within larger sample populations.

The strength of this investigation lies in its focus on a clinically significant yet understudied population whilst simultaneously examining anxiety and depressive disorders through comprehensive analysis of endocrine, metabolic and sociodemographic determinants. However, this study has several limitations. Firstly, the cross-sectional design precludes causal inference regarding the observed relationships. Secondly, as a single-centre study recruiting concurrently hospitalised patients, the sample may overrepresent those with more severe disease, thus limiting the generalisability of the findings to broader outpatient populations. Thirdly, although multiple confounders were adjusted for, unmeasured factors (e.g., specific menopausal symptoms, social support and detailed medication use) could influence outcomes. Finally, the multiple comparisons conducted without formal correction necessitate cautious interpretation of individual indicators.

## Conclusions

Postmenopausal women with T2DM and hypothyroidism are at high risk of anxiety and depressive symptoms, which are closely related to thyroid dysfunction, poor long-term blood glucose control and socio-economic factors. Based on the above findings and the limitations of the cross-sectional design and single-center sample of this study, the causal association and long-term prognostic impact of metabolic/endocrine indicators on emotional symptoms can be clarified through prospective longitudinal cohort studies in the future, and randomized controlled trials can be carried out to verify the effectiveness of the endocrine-psychological integrated intervention model. At the same time, refined studies are carried out for special subgroups such as different BMI stratification, hypothyroidism types and low income, so as to provide a more solid evidence-based basis for early screening, individualized intervention and comorbidity management of emotional symptoms in this population.

## Availability of Data and Materials

All experimental data included in this study can be obtained by contacting the corresponding author if needed.
